# Endoscopic ultrasound-guided super-selective partial splenic embolization: New embolic material and multiple safety precautions

**DOI:** 10.1055/a-2127-4737

**Published:** 2023-08-23

**Authors:** Wei Wu, Qi Wang, Yubei Gu, Yunwei Sun, Dong Wang, Duowu Zou, Zhiyuan Wu

**Affiliations:** 1 Department of Gastroenterology, Shanghai Jiao Tong University School of Medicine Affiliated Ruijin Hospital, Shanghai, China; 2 Department of Interventional Radiology, Shanghai Jiao Tong University School of Medicine Affiliated Ruijin Hospital, Shanghai, China

Partial splenic embolization (PSE) has proven useful in the management of two of the common sequelae of portal hypertension: bleeding varices and hypersplenism 1 . A combination of PSE and endoscopic therapy tends to further decrease the risk of variceal bleeding 2 . The authors have focused on enhancing the safety of endoscopic-ultrasound (EUS)-guided PSE 3 through super-selection and controlled embolization of the lower pole branch of the splenic artery and its supply area, utilization of a thinner needle, and double validation of the punctured artery. The delivery process of embolic material has been simplified as well ( Video 1 ).

**Video 1** Endoscopic ultrasound-guided super-selective partial splenic embolization: New embolic material and multiple safety precautions.


A 62-year-old man with alcoholic cirrhosis and portal hypertension benefitted from this strategy. He had an episode of acute esophageal variceal bleeding and was successfully managed by endoscopic therapy. During follow-up, the patient displayed residual varices and exacerbation of hypersplenism including decreased platelet and white blood cell (WBC) count, chronic fatigue, and skin bruising. Preoperative lab results showed a platelet count of 39 × 10 ^9^ /L, WBC count of 1.57 × 10 ^9^ /L, and total serum bilirubin of 66 μmol/L.

The procedure was performed under general anesthesia. With reference to coronal reconstruction computed tomography (CT) images, the lower pole branch of the splenic artery extending distally (caudally) to the probe was targeted to reduce post-procedural side effects ( Fig. 1 a ). Fine needle aspiration with a 22-gauge needle helped minimize bleeding risk. The successful puncture was confirmed by EUS-guided fine-needle-injection-based angiography with iohexol and contrast echoangiography with SonoVue ( Fig. 1 b ); in both methods contrast agent flow was shown directed to the splenic parenchyma. A 1:2 mixture of N-butyl-2-cyanoacrylate and lipiodol offers moderate fluidity and helps reduce regurgitation and excessive embolization. A total of 0.8 ml of diluted glue was injected under X-ray fluoroscopy and the needle was withdrawn without flushing ( Fig. 1 c ). The patient’s residual esophageal varices were concurrently managed by endoscopic ligation. He received empiric antibiotics for 2 weeks post-procedure. Although he experienced a fever, his overall condition remained satisfactory. Enhanced CT on day 5 revealed a 39.9 % embolization of spleen parenchyma without complications such as abscess, hematoma, or exacerbated portal vein thrombosis ( Fig. 1 d, e ) 4 . The patientʼs platelet count increased to 68 × 10 ^9^ /L on day 7 and he was discharged. At the 5-month follow-up, the patient exhibited a platelet count of 74 × 10 ^9^ /L, WBC count of 3.23 × 10 ^9^ /L, and improved liver function 1 , as evidenced by a decrease in total bilirubin to 45.2 μmol/L. The patient’s symptoms were relieved and he was free from further gastrointestinal bleeding in a total follow-up of 8 months to date.

**Fig. 1:**
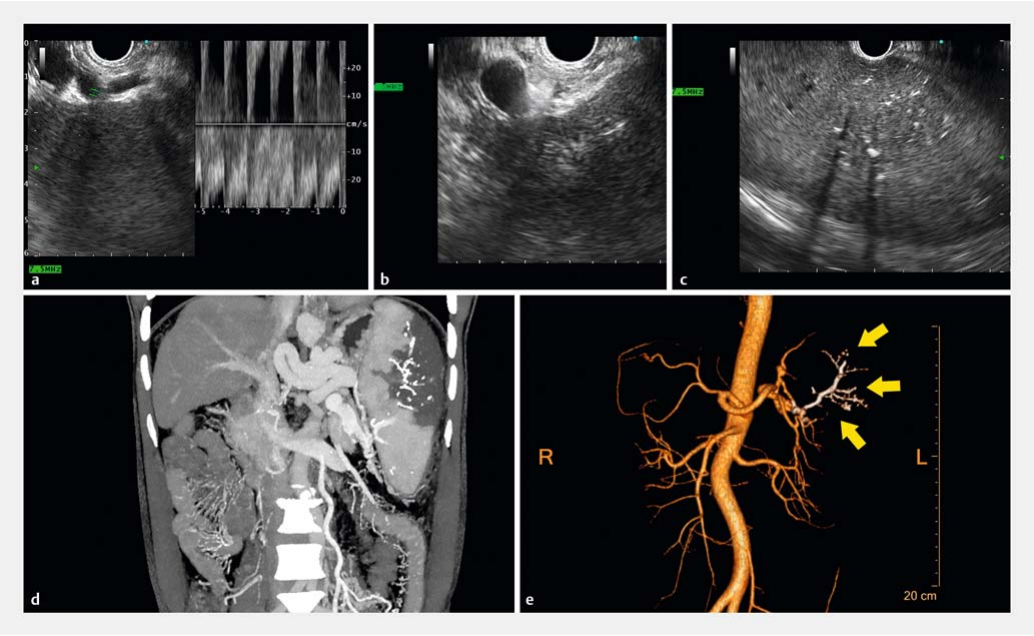
Endoscopic ultrasound (EUS)-guided super-selective partial splenic embolization. **a** Linear EUS was used to discern the lower pole branch of the splenic artery. Pulsed wave imaging demonstrated pulsating arterial waves. **b** The lower pole branch was punctured with a 22-gauge fine-needle aspiration (FNA) needle. Acoustic contrast agent was directly injected into the needle, and EUS revealed synchronized enhancement of the corresponding spleen parenchyma, indicating successful target artery puncture. **c** After embolization, EUS showed multiple hyper-echoic patches in the spleen parenchyma, with some leaving acoustic shadows behind. **d** Enhanced computed tomography (CT) confirmed a non-enhanced sector region in the lower lateral spleen, indicating splenic infarction induced by successful super-selected partial splenic embolization (PSE). Lipiodol deposition was shown as high-density substances inside the non-enhancement area. **e** 3D-reconstruction of abdominal arteries revealed the embolized lower pole branch and its sub-branches. Yellow arrows: polymerized glue with lipiodol.

Endoscopy_UCTN_Code_TTT_1AS_2AC

## Notes

Endoscopy E-Videos

https://eref.thieme.de/e-videos
*E-Videos* is an open access online section of the journal *Endoscopy* , reporting on interesting cases and new techniques in gastroenterological endoscopy. All papers include a high-quality video and are published with a Creative Commons CC-BY license. Endoscopy E-Videos qualify for HINARI discounts and waivers and eligibility is automatically checked during the submission process. We grant 100% waivers to articles whose corresponding authors are based in Group A countries and 50% waivers to those who are based in Group B countries as classified by Research4Life (see: https://www.research4life.org/access/eligibility/ ). This section has its own submission website at https://mc.manuscriptcentral.com/e-videos
